# Non-pharmacological treatment gap preceding surgical consultation in thumb carpometacarpal osteoarthritis - a cross-sectional study

**DOI:** 10.1186/s12891-019-2567-3

**Published:** 2019-04-30

**Authors:** Else Marit Holen Gravås, Anne Therese Tveter, Randi Nossum, Ruth Else Mehl Eide, Åse Klokkeide, Karin Hoegh Matre, Monika Olsen, Øyvor Andreassen, Nina Østerås, Ida Kristin Haugen, Ingvild Kjeken

**Affiliations:** 1 Department of Rheumatology, National Advisory Unit on Rehabilitation in Rheumatology, Diakonhjemmet Hospital, PO Box 23, Vinderen, N-0319 Oslo, Norway; 2 Department of Occupational therapy, Prosthetics and Orthotics, Faculty of Health Sciences, Oslo Metropolitan University, PO Box 4, St. Olavs plass, N- 0130 Oslo, Norway; 3 Department of Physiotherapy, Faculty of Health Sciences, Oslo Metropolitan University, PO Box 4, St. Olavs plass, N- 0130 Oslo, Norway; 4 Department of Rheumatology, St. Olavs Hospital, PO Box 3250, Sluppen, N-7006 Trondheim, Norway; 5 Department of Rheumatology, Haukeland University Hospital, PO Box 1400, N-5504 Bergen, Norway; 6 Haugesund Rheumatism Hospital, PO Box 2175, N-5504 Haugesund, Norway; 7 Department of Rheumatology, Diakonhjemmet Hospital, PO Box 23, Vinderen, N-0319 Oslo, Norway

**Keywords:** Hand osteoarthritis, Surgery, Interventions, Function, Occupational therapy

## Abstract

**Background:**

Osteoarthritis (OA) in the thumb carpometacarpal joint (CMCJ) is a prevalent disease which may lead to structural damage, severe pain and functional limitations. Evidence-based treatment recommendations state that all patients with hand OA should be offered non-pharmacological treatment. Surgery should be considered only when other treatment has proven insufficient in relieving pain. The purpose of this study was to investigate prior treatment and characteristics of patients referred to specialist health care surgical consultation due to CMCJ OA. The study includes exploring differences in pain and function between referred and non-referred hand, between men and women, and between patients with and without OA affection of other finger joints than CMCJ.

**Methods:**

Patients in this cross-sectional study reported prior non-pharmacological treatment for CMCJ OA. Patient demographics, disease and functional variables were assessed based on hand radiographs, patient-reported and observer-based outcome measures. Differences in pain and function between referred and non-referred hand, men and women, and between patients with and without additional affection of finger joints other than CMCJ, were analysed using Paired-samples T-tests, Wilcoxon Signed Rank, or Chi-Square tests.

**Results:**

One hundred and eighty patients were included. The mean age was 63 years and 79% were women. Only 21% reported having received non-pharmacological treatment before referral to surgical consultation. The results show a statistically significant worse function for referred hands, women and involvement of additional interphalangeal joints. Most patients reported no pain or mild pain in their referred hand.

**Conclusions:**

The results of this study show a non-pharmacological treatment gap in OA care. Most patients report no pain or mild pain, and that they had not received non-pharmacological treatment prior to being referred to CMCJ OA surgical consultation**.** The results furthermore show that CMCJ OA negatively affects all aspects of function. Strategies need to be developed to improve OA care, including educating general practitioners in evidence-based treatment recommendations and in the assessment of hand pain, and encourage the routine referral of patients with symptomatic hand OA to occupational therapy before considering surgery.

## Background

Osteoarthritis of the hand (HOA) is a highly prevalent disease that can potentially lead to pain, joint stiffness, reduced pinch and grip strength, impaired activity performance and can have a negative effect on work and quality of life [1–3]. In the Framingham cohort, radiographic HOA in at least one joint was observed in 44% of the women and 38% of the men between 40 and 84 years, while 14 and 7% of women and men, respectively, had symptomatic HOA in at least one joint. The thumb carpometacarpal joint (CMCJ) is one of the most commonly affected joints, and the observed prevalence of radiographic osteoarthritis (OA) in the CMCJ was 33% for women and 30% for men in the same study [4]. The thumb has a prominent role in hand functioning. Whether CMCJ OA is associated with more pain and disability than OA in other finger joints is a debated point [5–7].

There is currently no cure for OA. Non-pharmacological interventions, which include assistive devices, orthoses, hand exercise and patient education, are recommended as a core treatment for all patients with HOA. Surgery should only be considered for patients with structural abnormalities when other treatment modalities have not been sufficiently effective in relieving pain [8, 9]. Results from studies of hip and knee OA indicate that there is a treatment gap in OA care, as many patients do not receive recommended conservative treatment before being referred to orthopaedic surgery [10]. Little is, however, known about functional limitations and about the treatment received by patients with HOA before being referred to CMCJ OA surgical consultation. Studies have been carried out that compare HOA patients with and without CMCJ OA [5, 6]. There is, however, a lack of research that compares the symptoms and functional limitations in patients with isolated CMCJ OA with patients with a combination of CMCJ OA and OA in the interphalangeal joints (IPJ) of the hand.

The aim of this study was to investigate prior treatment and characteristics of patients referred to surgical consultation in specialist health care due to CMCJ OA. The study explores differences in pain and function between the referred and non-referred hand, between men and women, and between patients with and without OA affection (clinical nodes and pain) of finger joints besides CMCJ.

## Methods

### Study design

The study had a cross-sectional design, using baseline data from a multicentre randomised controlled trial (RCT) [11] (Trial registration: NCT01794754). The main aim of the RCT was to examine whether occupational therapy during the waiting period before surgical consultation could reduce or delay the need for CMCJ surgery.

### Study sample

Eligible participants were general practitioner (GP) referrals to CMCJ OA surgical consultation at three hospitals in Norway. The hospitals were the department of orthopaedics, St. Olav’s University Hospital, Trondheim, the department of plastic surgery, Haukeland University Hospital, Bergen and the department of rheumatology and orthopaedics, Haugesund Rheumatism Hospital, Haugesund. Patients unable to speak Norwegian or with cognitive dysfunctions were excluded.

### Data collection

Local coordinators at the three hospitals sent information about the study to patients on lists of surgical consultation referrals. Patients interested in participating responded using a pre-stamped envelope or by calling the coordinator. The coordinator then booked a baseline assessment appointment. At the baseline assessment, patients were screened for eligibility, and the written informed consent was collected before further assessment.

### Variables

The International Classification of Functioning, Disability and Health (ICF) model was used to categorise variables [12]. Personal factors (Table 1) included age, gender, marital status, work status, education level and hand dominance. Disease variables included current pharmacological therapy, comorbidities (high blood pressure/angina, infarction, other coronary heart disease/asthma, bronchitis, other lung disease/allergy, hay fever, eczema/sciatica/brain haemorrhage, stroke/cancer/neurological disease of the brain or nerve tissue/diabetes/ metabolic disorder/mental disorder/kidney disease/liver disease/ulcers or other stomach disorders/anaemia or other blood disease) (yes/no response), current co-existing diagnoses affecting the hands, previous injury and hand surgery (yes/no response), which hand(s) the patient was referred to surgical consultation for (left/right/both), and previous treatment for HOA.

**Table 1:** Personal factors and disease variables in 180 patients referred to surgical consultation due to carpometacarpal osteoarthritis

	Total (*n* = 180)	St. Olav’s Hospital (*n* = 81)	Haukeland University Hospital (*n* = 74)	Haugesund Rheumatism Hospital (*n* = 25)	*P* value
Age, years, mean (SD)	63.0 (7.6)	62.4 (7.6)	63.4 (7.3)	64.0 (8.7)	.61
Women, n (%)	142 (79)	71 (88)	53 (72)	18 (72)	.03
Living alone, n (%)	35 (19)	20 (25)	14 (19)	1 (4)	.07
Employed, n (%)	91 (51)	42 (52)	37 (50)	12 (48)	.94
Education more than 12 years, n (%)	63 (35)	29 (36)	26 (35)	8 (32)	.94
Hand dominance, right, n (%)	168 (93)	74 (91)	70 (95)	24 (96)	.61
Comorbidities, yes, n (%)	117 (65)	50 (62)	52 (70)	15 (60)	.46
Symptom duration, years, median (IQR)	5 (2,10)	5 (3,13)	4 (2,10)	2 (1,7)	.51
Previous hand surgery, yes, n (%)	36 (21)	11 (14)	20 (27)	5 (29)	.10
Referred for hand surgery					
in left hand, n (%)	53 (29)	27 (33)	21 (28)	5 (20)	
in right hand, n (%)	48 (27)	19 (24)	23 (31)	6 (24)	
in both hands, n (%)	79 (44)	35 (43)	30 (41)	14 (56)	.53

Numbers are reported as median and interquartile range (IQR), number and proportion (%), or mean and standard deviation (SD)

Differences between the three hospitals are analysed with the One-Way between groups ANOVA or Kruskal-Wallis test in continuous variables and Chi-square test in categorical variables, and reported with *p*-values

Body structures (Tables 2, 3 and 4) included: 1) severity of radiographic CMCJ OA classified using a modified Kellgren-Lawrence grade (KLG) scale (grade 0–4, 0 = no CMCJ OA) [4], 2) absence/presence of radiographic CMCJ subluxation [13], 3) ratio of radiographic CMCJ subluxation on frontal hand radiographs [14] and 4) number of finger joints with clinical nodes in distal interphalangeal joints (DIPJ), proximal interphalangeal joints (PIPJ) and the thumb interphalangeal joint (IP) (0–9 IPJ on each hand) [15]. The presence of clinical CMCJ subluxation and the extent of radial subluxation were measured using a digital calliper in the Osirix software by a doctor (IKH) experienced in reading hand radiographs.

OA affection in IPJ was defined as at minimum one finger joint with clinical nodes plus pain [16]. Radiographs were not taken of non-referred hands. No information was therefore available on radiographic severity and subluxation ratio of the CMCJ of these hands.

All body function measurements (Tables 2, 3 and 4) were carried out by experienced occupational therapists (RN, SD, REME, ÅK, KHM, MO). Maximal grip and pinch strength were measured in Newton using the Grippit electronic instrument. A standard test procedure was followed and normative measurement data is available [17]. Pain at rest and pain following grip and pinch strength measurements were self-reported by patients using Numeric Rating Scales (NRS) 0–10 (0 = no pain). The number of painful finger joints that were examined includes metacarpophalangeal joints (MCPJ), IP, PIPJ and DIPJ (0–14 joints on each hand). Flexion deficit of the 2nd to 5th fingers were recorded in millimetres as the distance between the proximal palmar crease to the distal point of each finger. This was summarised and computed as one variable for each hand. Range of motion was measured in degrees for thumb IPJ and MCPJ using a goniometer. Active palmar abduction of the thumb and active abduction of CMCJ were measured in degrees using the Pollexograph® and according to the procedures of de Kraker and colleagues [18].

Patient reported activity performance and participation (Tables 3 and 4) were recorded using the Measure of Activity Performance of the Hand (MAP-Hand, score 1 to 4, 1 = no activity problems) [19] and QuickDASH; the measurement of Disability of Arm, Shoulder and Hand (score 0–100, 0 = no disability) [20].

### Data analysis and statistics

Numbers and percentages are reported for categorical variables. Mean and standard deviation (SD) if normally distributed, or median and interquartile range (IQR) if skewed is reported for continuous variables.

The One-Way between groups ANOVA or Kruskal-Wallis test was used to examine differences in continuous variables between study patients referred to the three hospital departments. The Chi-square test was used to compare categorical variables. Body structure and body function differences between the referred and non-referred hand (for those with unilateral referral) and left and right hand (for those referred for both hands, bilateral referral) were assessed using the Paired-samples T-test if normally distributed, Wilcoxon Signed Rank test if skewed, or Chi-Square test if categorical. In addition to these variables, activity and participation were used when assessing differences between women and men and between different OA phenotypes (isolated CMCJ OA vs. CMCJ OA plus IPJ OA). The dependent variables in these comparisons were the values for the referred hand, or the mean of both hands for those with bilateral referral. Continuous variables were examined using the Independent-samples T-test or Mann-Whitney U test. Categorical variables were assessed using the Chi-square test. A *p*-value ≤0.05 was considered statistically significant. The NRS pain score cut off points were 1–4 for mild pain, 5–6 for moderate pain and ≥ 7 for severe pain [21, 22]. Each of the three pain variables are also reported as continuous variables. All analyses were performed using IBM SPSS software (version 21).

## Results

One hundred and eighty patients were included in the study (Table 1). The mean (SD) age was 63 (7.6) years and 142 (79%) were women. Eighty-three (46%) used analgesics, 63 (35%) used a Non Steroid Anti Inflammatory Drug (NSAIDs), 31 (17%) used a combination of analgesics and NSAIDs and 15 (8%) used Glucosamine. Self-reported comorbidities were present in 104 (64%) of the patients. Forty-four patients (24%) reported co- or pre-existing comorbidities or injuries of the hand, of which three (2%) reported current carpal tunnel syndrome. Additionally, 15 (8%) reported previous carpal tunnel syndrome surgery. Median (IQR) symptom duration was 5 (2 to 10) years. Only 37 (21%) self-reported having consulted an occupational therapist or physiotherapist before being referred to surgical consultation. These patients had statistically significant more pain at rest, lower max grip strength, less range of motion in IPJs and MCPJs, less thumb palmar abduction and also had longer symptom duration in years compared to patients with no prior non-pharmacological treatment. Nineteen (11%) had previously consulted a rheumatologist. There were no statistically significant differences between the patients of the three hospitals, except for a slightly higher proportion of women from St. Olav’s Hospital, Trondheim. Most patients (*n* = 101, 56%) were referred for unilateral surgery, 53 (29%) for left hand and 48 (27%) for right hand surgery, whereas 79 (44%) were referred for bilateral surgery. Sixty-three (35%) patients also had OA affection in IPJs in addition to CMCJ OA.

### Referred versus non-referred hand

The unilateral referral group had consistently better function in the non-referred hand compared to the referred hand (Table 2), with statistically significant differences in all body functions except for flexion deficit of the 2nd to 5th fingers. Forty-three (24%) of unilateral referral patients were referred for the dominant hand and 58 (32%) for the non-dominant hand.

**Table 2:** Unilateral referral and bilateral referral in 180 patients with carpometacarpal osteoarthritis

	Unilateral referral (*n* = 101)			Bilateral referral (*n* = 79)		
	Referred hand	Non-referred hand	*P* value	Left	Right	*P* value
*Body structure*						
Radiographic CMCJ OA severity (0–4, 0 = no CMCJ OA), median (IQR)	3 (3,4)	–	–	3 (3,4)	3 (2,4)	.16
Presence of clinical CMCJ subluxation, n (%)	65 (64)	–	–	46 (58)	38 (48)	.02
Radiographic CMCJ subluxation ratio, mean (SD)	0.51 (0.10)	–	–	0.50 (0.10)	0.48 (0.11)	.04
Number of finger joints with clinical nodes (0–9 joints), median (IQR)	0 (0,2)	0 (0,1)	<.001	0 (0,2)	0 (0,2)	NA
*Body function*						
Pain at rest (0–10 scale), median (IQR)	3 (1,5)	0 (0,2)	<.001	3 (1,5)	2 (0,4)	.11
Pain following measure of grip strength (0–10, 0 = no pain), median (IQR)	4 (1,5)	0 (0,2)	<.001	3 (1,4)	2 (1,5)	.84
Pain following measure of pinch strength (0–10, 0 = no pain), median (IQR)	4 (1,6)	0 (0,2)	<.001	3 (2,5)	3 (1,5)	.33
Max grip strength, Newton (N), median (IQR)	159 (106,240)	214 (150,316)	<.001	159 (97,234)	177 (118,235)	.02
Max pinch strength, N, median (IQR)	29 (20,40)	37 (27,48)	<.001	30 (20,39)	29 (23,43)	.40
Grip strength referred hand (% of normal grip strength), mean (SD)	65 (25)	82 (26)	<.001	64 (27)	67 (29)	.33
Pinch strength referred hand (% of normal pinch strength), mean SD	60 (25)	76 (29)	<.001	61 (27)	65 (27)	.10
Number of painful finger joints (MCPJ, IP, PIPJ, DIPJ: 0–14 joints), median (IQR)	1 (0,4)	1 (0,4)	NA	2 (1,5)	3 (1,5)	.26
Flexion deficit 2nd-5th fingers, mm, median (IQR)	0 (0,0)	0 (0,0)	.09	0 (0,3)	0 (0,0)	.40
Range of motion thumb IP, degrees, median (IQR)	70 (58,76)	75 (65,80)	<.001	70 (62,80)	70 (60,70)	.50
Range of motion MCP1, degrees, median (IQR)	50 (42,56)	55 (45,65)	<.001	50 (40,60)	50 (42,60)	.42
Palmar abduction thumb, degrees, median (IQR)	46 (40,55)	56 (47,62)	<.001	50 (42,58)	50 (42,58)	.60
Abduction CMCJ, degrees, median (IQR)	36 (30,42)	42 (34,49)	<.001	37 (32,44)	39 (30,44)	.55

Numbers are reported as median and interquartile range (IQR), number and proportion (%), or mean and standard deviation (SD). Radiographic CMCJ OA severity is classified using modified Kellgren-Lawrence scale (grade 0–4, 0 = no CMCJ OA). Pain is self-reported using Numeric Rating Scales (NRS 0–10, 0 = no pain). Grip and pinch strength is measured in Newton (N) using the Grippit electronic instrument. Flexion deficit is measured in millimeters (mm). Range of motion is measured in degrees with goniometer. Palmar abduction thumb and CMCJ abduction is measured in degrees using the Pollexograph®

Differences between referred hand/non-referred hand and between left/right referral are analysed with Paired-samples t-test if normally distributed, Wilcoxon Signed Rank test if skewed, or Chi-Square test if categorical, and reported with p-values

*NRS* Numeric Rating Scale, *MCPJ* metacarpophalangeal joint, *IP* thumb interphalangeal joint, *PIPJ* proximal interphalangeal joints, *DIPJ* distal interphalangeal joints, *MCP1* thumb metacarpophalangeal joint, *NA* not applicable to calculate *p*-value because of many ties

**Fig. 1:**
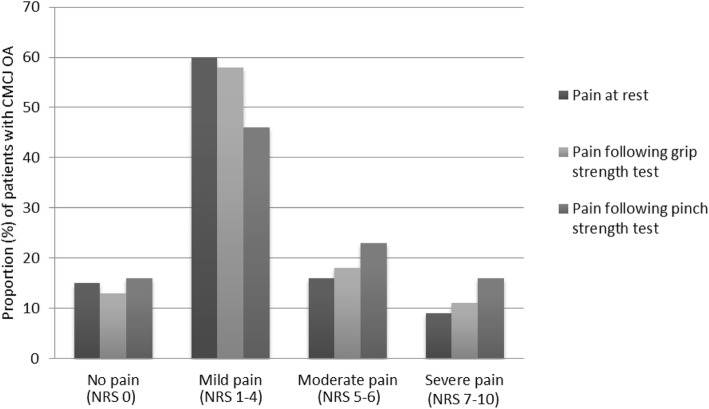
Pain in referred hand(s) in 180 patients referred for surgical consultation due to carpometacarpal osteoarthritis. *NRS* Numeric Rating Scale (0–10, 0 = no pain). *CMCJ OA* thumb carpometacarpal joint osteoarthritis

The majority of patients reported no pain (13–16%) or mild pain (46–60%). Median (IQR) pain levels were 3.0 (1 to 4) at rest, 3.0 (2 to 5) following grip strength measurement and 3.5 (2 to 6) following pinch strength measurement (Fig. 1).

There were statistically significant more patients with CMCJ subluxation in the left hand than in the right (Table 2) and subluxation was statistically significant more severe in the left than the right hand. There were no differences between left and right hand body functions for bilateral referral patients, other than statistically significant lower left hand max grip strength than the right hand. There were also no statistically significant differences in activity and participation between unilateral and bilateral referrals, as shown by MAP-Hand and QuickDASH scores.

### Gender differences

Radiographic severity in the CMCJ was similar in men and women. Women, however, had statistically significant more joints with interphalangeal nodes (Table 3). Women also had statistically significant lower grip and pinch strength, and more painful finger joints than the men in the study. Compared to normative values, women in our study showed lower percentage of normal grip and pinch strength as opposed to the men. Women also reported statistically significant more activity limitations and participation restrictions than men, as measured by MAP-Hand and QuickDASH.

**Table 3:** Gender differences in body structures, body functions and activity and participation in 180 patients with carpometacarpal osteoarthritis

	Women (*n* = 142)	Men (*n* = 38)	*P* value*
*Body structure*			
Radiographic CMCJ OA severity (0–4, 0 = no CMCJ OA), median (IQR)	3 (3,4)	3 (3,4)	.73
Presence of clinical CMCJ subluxation, n (%)	89 (63)	24 (63)	.88
Radiographic CMCJ subluxation ratio, mean (SD)	0.50 (0.10)	0.50 (0.08)	.76
Number of finger joints with clinical nodes (0–9 joints), median (IQR)	0 (0,2)	0 (0,0)	.01
*Body function*			
Pain at rest (0–10, 0 = no pain), median (IQR)	3 (1,5)	2 (1,4)	.31
Pain following measure of grip strength (0–10, 0 = no pain), median (IQR)	3 (2,5)	3 (1,5)	.72
Pain following measure of pinch strength (0–10, 0 = no pain), median (IQR)	3 (2,5)	4 (2,6)	.81
Max grip strength, Newton (N), median (IQR)	145 (62)	336 (95)	<.001
Max pinch strength, N, median (IQR)	27 (11)	50 (20)	<.001
Grip strength referred hand (% of normal grip strength), mean (SD)	59 (23)	83 (25)	<.001
Pinch strength referred hand (% of normal pinch strength), mean SD	59 (24)	69 (28)	.04
Number of painful finger joints (MCPJ, IP, PIPJ, DIPJ: 0–14 joints), median (IQR)	3 (1,5)	1 (0,2)	<.001
Flexion deficit 2nd-5th fingers, mm, median (IQR)	0 (0,6)	0 (0,0)	.62
Range of motion thumb IP, degrees, median (IQR)	70 (61,78)	68 (60,76)	.48
Range of motion MCP1, degrees, median (IQR)	50 (42,59)	50 (40,56)	.99
Palmar abduction thumb, degrees, median (IQR)	48 (42,56)	47 (38,55)	.45
Abduction CMCJ, degrees, median (IQR)	36 (31,42)	36 (30,44)	.94
*Activity and participation*			
Activity performance (1–4, 1 = no activity problems), mean (SD)	2.0 (0.4)	1.7 (0.4)	<.001
Function and symptoms arm, shoulder and hand (0–100, 0 = no disability), mean (SD)	38.6 (16.4)	30.4 (15.6)	.01

Numbers are reported as median and interquartile range (IQR), number and proportion (%), or mean and standard deviation (SD). Radiographic CMCJ OA severity is classified using modified Kellgren-Lawrence scale (grade 0–4, 0 = no CMCJ OA). Pain is self-reported using Numeric Rating Scales (NRS 0–10, 0 = no pain). Grip and pinch strength is measured in Newton (N) using the Grippit electronic instrument. Flexion deficit is measured in millimetres (mm). Range of motions is measured in degrees with goniometer. Palmar abduction thumb and CMCJ abduction is measured in degrees using the Pollexograph®. Activity performance is measured with mean score of MAP-Hand (1–4, 1 = no activity problems). Function and symptoms arm, shoulder and hand is measured using sum score of QuickDASH (0–100, 0 = no disability)

*Values for body structure and body function variables are values for referred hand in patients referred for one hand, and mean of both hands in patients referred for both hands. Differences between women and men are analysed with Paired-samples t-test if normally distributed, Wilcoxon Signed Rank test if skewed, or Chi-Square test if categorical, and reported with *p*-values

*NRS* Numeric Rating Scale, *MCPJ* metacarpophalangeal joints, *IP* thumb interphalangeal joint, *PIPJ* proximal interphalangeal joints, *DIPJ* distal interphalangeal joints, *MCP1* thumb metacarpophalangeal joint

### Isolated CMCJ OA versus CMCJ OA plus IPJ OA

Patients with CMCJ OA plus IPJ OA had statistically significant more pain, lower grip and pinch grip strength and less joint mobility than those with isolated CMCJ OA. Those with CMCJ OA plus IPJ OA also reported more activity limitations and participation restrictions than patients with isolated CMCJ OA (Table 4).

**Table 4:** Differences in patients with isolated carpometacarpal osteoarthritis versus patients with osteoarthritis also in other finger joints

	Isolated CMCJ OA (*n* = 117)	CMCJ plus IPJ OA (*n* = 63)	*P* value*
*Body structure*			
Radiographic CMCJ OA severity (0–4, 0 = no CMCJ OA), median (IQR)	3 (3,4)	3 (3,4)	.82
Presence of clinical CMCJ subluxation, n (%)	79 (73)	34 (59)	.08
Radiographic CMCJ subluxation ratio, mean (SD)	0.50 (0.09)	0.48 (0.11)	.14
Number of finger joints with clinical nodes (0–9 joints), median (IQR)	0 (0,0)	3 (2,4)	<.001
*Body function*			
Pain at rest (0–10, 0 = no pain), median (IQR)	3 (1,4)	4 (2,5)	.05
Pain following measure of grip strength (0–10, 0 = no pain), median (IQR)	3 (1,5)	4 (2,5)	.41
Pain following measure of pinch strength (0–10, 0 = no pain), median (IQR)	4 (1,6)	4 (2,5)	.87
Max grip strength, Newton (N), median (IQR)	176 (112,274)	152 (101,196)	.03
Max pinch strength, N, median (IQR)	31 (22,43)	27 (19,34)	.02
Grip strength referred hand (% of normal grip strength), mean (SD)	66 (26)	61 (23)	.23
Pinch strength referred hand (% of normal pinch strength), mean SD	63 (26)	57 (22)	.12
Number of painful finger joints (MCPJ, IP, PIPJ, DIPJ: 0–14 joints), median (IQR)	1 (0,3)	5 (3,7)	<.001
Flexion deficit 2nd-5th fingers, mm, median (IQR)	0 (0,0)	0 (0,41)	<.001
Range of motion thumb IP, degrees, median (IQR)	71 (64,80)	65 (55,75)	.02
Range of motion MCP1, degrees, median (IQR)	50 (41,60)	49 (42,55)	.56
Palmar abduction thumb, degrees, median (IQR)	49 (42,58)	46 (39,51)	.01
Abduction CMCJ, degrees, median (IQR)	37 (32,44)	35 (29,40)	.02
*Activity and participation*			
Activity performance (1–4, 1 = no activity problems), mean (SD)	1.9 (0.4)	2.1 (0.4)	<.001
Function and symptoms arm, shoulder and hand (0–100, 0 = no disability), mean (SD)	34.3 (15.7)	41.6 (17.0)	.01

Numbers are reported as median and interquartile range (IQR), number and proportion (%), or mean and standard deviation (SD). Radiographic CMCJ OA severity is classified using modified Kellgren-Lawrence scale (grade 0–4, 0 = no CMCJ OA). Pain is self-reported using Numeric Rating Scales (NRS 0–10, 0 = no pain). Grip and pinch strength is measured in Newton (N) using the Grippit electronic instrument. Flexion deficit is measured in millimetres (mm). Range of motions is measured in degrees with goniometer. Palmar abduction thumb and CMCJ abduction is measured in degrees using the Pollexograph®. Activity performance is measured with mean score of MAP-Hand (1–4, 1 = no activity problems). Function and symptoms arm, shoulder and hand is measured using sum score of QuickDASH (0–100, 0 = no disability)

*Values for body structure and body function variables are values for referred hand in patients referred for one hand, and mean of both hands in patients referred for both hands. Differences between OA affection in isolated CMCJ and OA affection in CMCJ plus in IPJ are analysed with Paired-samples t-test if normally distributed, Wilcoxon Signed Rank test if skewed, or Chi-Square test if categorical, and reported with p-values

*NRS* Numeric Rating Scale, *MCPJ* metacarpophalangeal joints, *IP* thumb interphalangeal joint, *PIPJ* proximal interphalangeal joints, *DIPJ* distal interphalangeal joints, *MCP1* thumb metacarpophalangeal joint

## Discussion

All of the patients in this study were referred to specialist health care surgical consultation due to CMCJ OA. Most of these patients reported no pain or mild pain in the referred hand. Only a minority had received the recommended first-line non-pharmacological treatment, the remainder being referred directly to surgical consultation by their GP. The results furthermore show that unilateral referral patients consistently reported better function in the non-referred hand, and that there were only minor differences between left and right hand in bilateral referral patients. Gender differences include women having poorer scores than men in most aspects of function. Patients with both CMCJ OA and IPJ OA furthermore reported more severe symptoms and functional limitations than those with isolated CMCJ OA.

Pain is the predominant symptom that leads patients with OA to contact their GP [23], and the main indication for CMCJ surgery is to reduce pain and increase function [9]. There is, however, growing evidence that non-pharmacological interventions such as patient education, hand exercises, orthoses and assistive devices reduce pain and improve function in HOA patients [24–26]. Complications, repeated surgery and substantial periods of time off work are furthermore frequently reported after CMCJ surgery [27, 28]. Accordingly, the European League Against Rheumatism recommendations state that surgery should only be considered for patients with structural abnormalities when other treatment modalities have not been sufficiently effective in relieving pain [29]. Still, the majority of patients in our study reported no pain or mild pain. This may be an indication that GP consultation quality needs to be improved for this patient group. One strategy may be to inform GPs about simple and timesaving procedures, such as using a patient’s pain-level rating to inform the referral to surgical consultation decision.

In line with studies on hip and knee OA [10], only a minority of the patients in our study had consulted an occupational therapist or physiotherapist before surgical consultation. One reason for this may be that many GPs are not sufficiently up to date on treatment recommendations and the beneficial effects of non-pharmacological interventions. GPs therefore refer patients to the treatment they are familiar with, namely surgery, a hypothesis supported by findings from qualitative studies. Patients with HOA reported, in these studies, a lack of support and information on management of their condition and that other non-pharmacological treatments were rarely offered or tried before referral to surgical consultation [30–32]. Other studies indicate that increased workload and lack of time and experience are key barriers to GPs engaging in optimal OA care [33, 34]. A strategy that has been proven to be effective in improving the quality of care is the implementation of a primary care-based OA model [35]. This includes an enhanced OA consultation by a GP, follow-up consultations with an OA trained nurse and access to a broader multidisciplinary team including occupational therapists and physiotherapists [36, 37]. Another approach is to build alliances with patient organisations and use social media to enhance and disseminate knowledge about disease consequences and effective self-management strategies for those with HOA. Educated patients can inform GPs about and request available and effective treatment options. This can break the vicious circle of patients not consulting health professionals, thereby concealing their problems and the lack of treatment services and disparities between the services offered to different patient groups [30].

As expected, unilateral referral patients in this study reported consistently worse function in their referred hand, and women had lower hand strength and more functional limitations than men. The gender differences are in line with previous studies of HOA patients [38], rheumatoid arthritis patients [39] and in the general population [40, 41]. The association between muscle weakness and functional limitations [38, 42] is well known. Strengthening exercises have, however, been found to improve hand function in HOA patients [25]. Women display lower muscle strength than men [43], and the functional consequences of muscle weakness therefore have a greater affect upon women. This emphasises that women with HOA should focus on exercises that improve strength and hand function.

Although results are somewhat ambiguous, previous research has shown that CMCJ OA and IPJ OA functional limitations are comparable [6] and that the ailment of patients with IPJ OA increase when they also suffer from CMCJ OA [5]. The current study suggests that additional IPJ OA involvement in patients with CMCJ OA worsens symptoms and functional limitations, indicating that involvement of more joints in the hand will cause more functional limitations, regardless of initial joint involvement. Future studies should explore whether there is a linear relationship between number of joints and degree of disability, with the increased number of affected joints leading to increasingly limited function regardless of the joint(s) involved, or if there are certain patterns of involvement that are more disabling than others.

Our findings in general support the hypothesis that there is a treatment gap in OA care [44, 45]. Dziedzic and colleagues suggest that the quality of this care can be improved by taking factors outside the joint into consideration and by introducing a biopsychosocial model as an alternative to the medical approach. They furthermore suggest that occupational therapists play a crucial role in the care of HOA patients. Occupational therapists have been trained in approaches that take into consideration psychological, social, and environmental needs, and in methods to reduce activity limitations and participation restrictions caused by HOA [46]. Encouraging GPs to routinely refer patients with HOA to an occupational therapist as a first-line treatment may, therefore, be one way of ensuring better support for OA patients.

The availability of only referred hand radiographs represents a limitation of this study. This, however, only slightly limited the referred hand/non-referred hand comparison. A further limitation is the cross sectional design, which does not allow cause and effect relationship conclusions to be drawn. It should, however, be kept in mind when comparing pain and function of the referred hand versus non-referred hand, that the patients may also have OA in the non-referred hand. Some patients also had co-existing diseases, which can potentially affect self-reported outcome measurements. The use of analgesics and assistive devices were self-reported. This can have induced a recall bias. The strengths of the study include the large study sample, the thorough clinical and radiographic evaluation combined with patient self-reported data, and that patients were recruited from three different and geographically separate hospitals.

## Conclusion

The results of this study show that there is a non-pharmacological treatment gap preceding surgical consultation in CMCJ OA. Most patients referred to surgical consultation reported no pain or mild pain. They also had not received the recommended non-pharmacological treatment before being referred**.** The results further show that CMCJ OA negatively affected all aspects of function, especially in the referred hand, in women, and in those who also had IPJ OA. Strategies need to be developed to improve clinical practice. These include educating GPs in evidence-based treatment recommendations and in the assessment of hand pain, and to routinely refer those with symptomatic HOA to occupational therapy before considering surgery.

## Abbreviations

CMCJ: Thumb carpometacarpal joint
DIPJ: Distal interphalangeal joint
GP: General practitioner
HOA: Hand osteoarthritis
ICF: International Classification of Functioning, Disability and Health
IP: Thumb interphalangeal joint
IPJ: Interphalangeal joint
IQR: Interquartile range
KLG: Modified Kellgren-Lawrence grade
MCPJ: Metacarpophalangeal joint
NRS: Numeric Rating Scale
NSAIDs: Non Steroid Anti Inflammatory Drugs
OA: Osteoarthritis
PIPJ: Proximal interphalangeal joint
RCT: Randomized controlled trial
SD: Standard deviation
SPSS: Statistical Package for the Social Sciences
